# Impact of Milk on the Behavior and Toxicity of Nanosized SiO_2_ Particles during *In Vitro* Digestion Simulation

**DOI:** 10.1021/acsomega.5c03484

**Published:** 2025-06-12

**Authors:** Hafize Öz Elrai, Nazım Sergen Mısırlı, Esin Bayan Karakadılar, Seda Yildirim-Elikoğlu, Fahriye Ceyda Dudak

**Affiliations:** † Graduate School of Science and Engineering, 37515 Hacettepe University, Beytepe, 06800 Ankara, Turkey; ‡ Department of Food Engineering, 37515 Hacettepe University, Beytepe, 06800 Ankara, Turkey

## Abstract

Silicon dioxide nanoparticles (SiO_2_ NPs) are commonly ingested by humans via food products, yet their potential biological effects remain poorly understood. To address this knowledge gap, we conducted a study to investigate the effects of a real food matrix and *in vitro* digestion on the behavior and toxicity of SiO_2_ NPs. The 25, 100, and 300 nm SiO_2_ particles (Si25, Si100, and Si300, respectively) were incubated with milk to simulate real-world conditions. Protein corona layers with varying thicknesses were observed for each particle. The main components of these corona layers were α- and β-caseins, and they were more abundant than whey proteins. Bare and milk-incubated particles went through a realistic digestion simulation with simulated saliva (SSF), gastric (SGF), and intestinal (SIF) fluids. TEM images revealed that the corona structure remained until the end of the digestion simulation. Toxicity analyses showed that as SiO_2_ NPs decreased in size, they exhibited more toxic effects on Caco-2 cells, regardless of digestion simulation. The corona mitigated the cytotoxicity of Si25. Additionally, we observed that SiO_2_ NPs induced reactive oxygen species (ROS) production depending on the particle size and corona formation. These findings have implications for the safe use of SiO_2_ NPs in consumer products and highlight the need for further research into the mechanisms underlying NP-induced ROS production and cytotoxicity.

## Graphical abstract

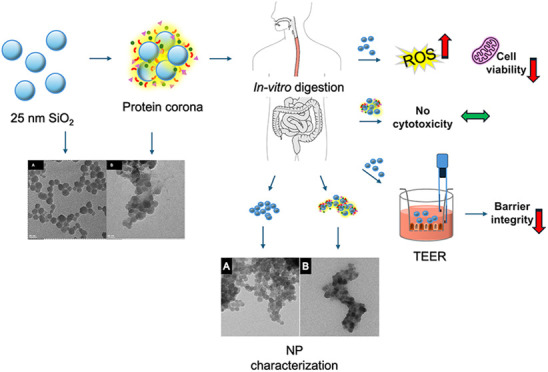

## Introduction

The growing global population has increased the demand for food worldwide. Various additives have been incorporated into the food industry to ensure optimal use and preservation of food products. Food additives are substances added to food products to enhance their shelf life, flavors, appearance, or texture. These additives play a crucial role in maintaining the quality and appeal of foods from production to consumption.

Food additives are regulated by health and food authorities, who review the available scientific literature to assess their safety and establish guidelines for their application. However, food additives are widely used, some are not considered entirely safe, and there are ongoing concerns about their potential health implications.^1,2^ Among these additives, SiO_2_ (E551/silica) is mainly used as an anticaking, antifoaming, coloring, and flavor adjunct in a variety of food products such as salt, flour, powdered soup, and coffee whiteners.^3^ Until recently, the acceptable daily intake (ADI) value for SiO_2_ (E551) was not specified. A reevaluation of SiO_2_ by the European Food Safety Authority (EFSA) revealed the need for further studies since the available toxicological data are limited in providing an ADI value.^2^ According to previous research, the average daily dietary intake of SiO_2_ was estimated to be in the range of 0.28–12.7 mg/kg bw.^4,5^ Although the food additive SiO_2_ has been reported to present as microsized aggregates and agglomerates, studies showed that nanosized SiO_2_ (<50 nm) can also be found in additive silica.^6^ Dekkers, Krystek, Peters, Lankveld, Bokkers, van Hoeven-Arentzen, Bouwmeester, and Oomen^4^ demonstrated that up to 33% of the silica in E551 has exterior sizes ranging from 10 to 200 nm. Furthermore, nanosized (<30 nm) SiO_2_ particles have been detected in various food products.^7^

There is growing concern about the potential health risks of NPs with their widespread usage and subsequent direct or indirect exposure to the human body. Potential toxicity of inorganic NPs, which is affected by several factors including particle size, shape, aggregation state, chemical composition, surface properties, and biocorona formation,^8^ has been documented. The small size and enhanced surface area of NPs lead to elevated chemical reactivity and penetration rates into the cells and tissues.^9^ NPs with different physicochemical and electrical properties have been reported to cause cytotoxicity and genotoxicity due to elevated ROS production and DNA damage in cells.^10^ Furthermore, exposure to inorganic NPs may cause accumulation in various tissues and organs in the long term.^11^

SiO_2_ NP exposure has been reported to be associated with increased ROS levels and altered protein expression in cells.^12^ On the other hand, Yun, Kim, You, Kim, Jang, Min, Kim, Chung, Jeong, Kang, and Che^13^ concluded that SiO_2_ did not cause any accumulation or toxicity in rats. The conflicting results regarding the toxicity of NPs can arise from variations in the characteristics of the particles. NP behavior and toxicity strongly depend on the surrounding medium.^8^

The potential of biomolecules adhering to the NP surface and forming a “biocorona” is a well-known phenomenon. Formation of the corona layer via electrostatic, hydrophobic, or steric interactions between NPs and biomolecules was reported to affect the surface characteristics of NPs, which might eventually alter the aggregation state, reactivity, and toxicity.^14,15^ It has been reported that when cells were exposed to titanium dioxide NPs (TiO_2_) in the presence of human serum proteins, they caused more cell damage than exposure to TiO_2_ alone.^16^ Besides serum proteins, studies also showed that the toxicity and behavior of NPs (concerning oral uptake) might be affected by the interactions with the molecules in foods.^15,17^ Because of the proteins that it contains, food matrices provide a favorable environment for NPs to form a biocorona. In our previous study, we examined the behavior and *in vitro* toxicity of different sized TiO_2_ NPs that interacted with a realistic food matrix, containing mixed protein groups, through a realistic digestion simulation.^15^ The study showed that bare particles caused greater cellular stress and ROS production compared with corona-coated particles. *In vitro* studies have determined that the corona formation on SiO_2_ NPs can affect NP uptake and cell death in endothelial tissue.^7^ One study compared two different *in vitro* digestion conditions and observed changes in SiO_2_ NP behavior that affected toxicity to cell lines. They found that NP transport across the cell monolayer and NP-induced cell death were lower with fed-state simulated digestion fluids than with fasting-state or buffer-only conditions.^18^ When oral uptake of SiO_2_ NPs is considered, interaction between NPs and the food matrix as well as GI system components seems to be crucial in determining the fate of particles. Therefore, toxicity studies involving models or real food matrices have gained great interest over the past few years.

This study aims to determine the effects of the real food matrix and digestion on agglomeration behavior, hard protein corona structures, and acute toxicity of SiO_2_ NPs at different sizes. Various dietary environments, including solid food matrices (e.g., meat, vegetables, grains), carbohydrate-rich processed foods (e.g., sugary beverages, bakery products), and lipid-based systems (e.g., anticaking agents), may influence nanoparticle behavior in distinct ways.^19,20^ Milk was selected as the model food matrix because of its common consumption and physicochemical characteristics, which closely resemble those of the FDA-recommended standard meal for drug bioavailability studies.^21^ SiO_2_ NPs are widely used in dairy-based powdered products (e.g., coffee creamers), making milk a suitable model. Milk contains a complex mixture of proteins, lipids, and minerals that contribute to nanoparticle stabilization, aggregation, and protein corona formation. Additionally, its composition closely mimics that of processed foods where SiO_2_ additives are frequently employed. The digestion of milk further affects the physicochemical transformations of nanoparticles, making it a relevant system for examining their behavior under physiological conditions. Within the scope of the study, changes in the properties of nanoparticles interacting with whole and skim milk and the toxic effects of nanoparticles on cell culture after the digestion simulation were investigated. The findings of this study reveal the importance of alterations in the properties and toxicity of SiO_2_ NPs following incubation with a real food matrix.

## Materials and Methods

### Materials

Whole milk (WM) and skim milk (SM) powders were supplied by Enka Dairy Co. (Konya, Turkey). Amorphous SiO_2_ nanoparticles (25 nm, 30% in water dispersion) (Si25) were purchased from Nanografi Nanotechnology A.S. (Ankara, Turkey). SiO_2_ nanoparticles of 100 nm (Si100) and 300 nm (Si300) were kindly provided by Kouroush Salimi (Ankara Yıldırım Beyazıt University, Turkey). These nanoparticles were synthesized using the sol–gel process (Stöber method) via the hydrolysis of tetraethyl orthosilicate (TEOS) in an ethanol medium.^22^ Dithiothreitol (DTT), used in electrophoresis, was obtained from Roche (Basel, Switzerland). Acrylamide, bis­(acrylamide), and coomassie brilliant blue were purchased from Bio-Rad (Hercules, CA, U.S.A.). All other reagents were purchased from Sigma-Aldrich Chemicals (St. Louis, MO, U.S.A.).

### Interaction of SiO_2_ NPs with Milk Samples

SiO_2_ NP stock solutions (42 mg/mL) were prepared in simplified simulated milk ultrafiltrate buffer (SMUB, pH 6.7, 0.02 M Tris base, 0.05 M NaCl 0.003 M CaCl_2_)^23^ and sonicated for 15 min at 35% power (650 W), 20–25 kHz in continuous mode before testing via JY92-IIN Ultrasonic Homogenizer (Hinotek, Ningbo, China). The crystalline structures of SiO_2_ NPs were characterized via XRD (PAN- alytical X’Pert3 Powder, Malvern Panalytical Ltd., England) by drying the NPs in TBS dispersion. The measurements with Cu Kα radiation (45 kV, 40 mA) were made using continuous scan mode in the range of 10–80° (2θ) (λ = 0.154 nm), with a scan step size of 0.01°.

Whole milk and skim milk powders were reconstituted in distilled water. In our previous study, we obtained the final composition and the acidity of whole milk (WM, pH 6.7 ± 0.1) and skim milk (SM, pH 6.7 ± 0.1) samples.^15^

Individual stock solutions of SiO_2_ (42 mg/mL) NPs were prepared in SMUB. The concentrations of NPs were determined based on the daily intake of 700 mg assuming an adult body weight of 70 kg. The concentration of 3.5 mg/mL for SiO_2_ represents the concentrations of NPs to be ingested when 200 mL of food is consumed.^4^ Additionally, lower and higher concentrations (1.75 and 7 mg/mL) were examined. The suspensions of NPs were incubated with WM and SM solution at room temperature for 1h with vigorous shaking. The same concentrations of NPs were incubated with SMUB as the control samples. Following the incubation period, the NP-protein corona was separated via centrifugation (4000*g* for 15 min). Subsequently, the pellet was washed three times with SMUB to remove any unbound or loosely bound proteins before the ex-situ analysis.

### *In Vitro* Digestion Simulation

In the simulation of *in vitro* digestion, only WM was used as a food matrix because it represents a realistic model for future assessments. The standardized static *in vitro* digestion system^24^ was selected for this research, given its reliability in replicating gastrointestinal environments and widespread adoption in the field. This model provides precise control over digestive variables, ensuring robust comparisons of the nanoparticle dynamics. The gastrointestinal environment (GI) was prepared following the methodology outlined by Sohal, Cho, O’Fallon, Gaines, Demokritou, and Bello^25^ with some modifications as described briefly in our previous study.^15^ After the SiO_2_ NPs were incubated with SMUB or WM for 1 h at room temperature, the suspensions were subjected to a sequential *in vitro* digestion using simulated saliva (SSF), gastric (SGF), and intestinal fluids (SIF). The standardized procedure described by previous studies.^15,24^ After the digestion process, samples were separated by centrifugation (4000*g* for 15 min) and washed with SMUB. In this study, we investigated the aggregation-dispersion state and the hydrodynamic diameters of SiO_2_ NPs in each simulated GI fluid, demonstrating the formation of a protein corona through further analysis.

### Characterization of SiO_2_ NPs and Protein Corona

The morphologies and aggregation states of bare NPs, NPs that interacted with milk samples, and digested NPs were investigated by TEM and ESEM imaging. TEM imaging was carried out using an FEI Tecnai G2 Spirit BioTwin electron microscope from Thermo Fisher Scientific, MA, USA. The samples were diluted 20 times with distilled water for optimal imaging. A 5 μL aliquot of the diluted sample was deposited onto a carbon-coated 400 mesh copper grid and air-dried before visualization. Samples were examined at different accelerating voltages of 15–20 and 30 kV with different magnifications (40,000× and 80,000×) with ESEM (FEI-Quanta 200 FEG-Thermo Fisher Scientific Co., Waltham, MA, USA). The samples were dried on an aluminum stub and sputter-coated with a 5 nm Au layer.

The hydrodynamic diameters and zeta potential values of NPs were determined through DLS measurements conducted using a Zetasizer Nano ZS from Malvern Instruments Ltd., Malvern, UK, equipped with disposable cuvettes at 25 °C. Before measurements, all samples were diluted 5-fold with SMUB to minimize the impact of particle reflection. The refractive indices of SMUB and NPs were set at 1.34 and 2.41, respectively. The hydrodynamic diameters were calculated using the Stokes–Einstein equation. In the case of zeta measurements, the Smoluchowski equation was applied since the dielectric constant of the solvent medium exceeds 70. The measurements were performed in triplicate, and the results were averaged, with ten measurements considered for each replication.

In order to identify the proteins adsorbed onto SiO_2_ NPs, one-dimensional sodium dodecyl sulfate polyacrylamide gel electrophoresis (SDS-PAGE) was performed. Milk interacted, and digested NPs samples were centrifuged at 4000*g* for 15 min. Subsequently, pellets were resuspended in the SDS sample buffer (125 mM DTT). After resuspension, samples were denatured at 95 °C for 20 min. Postdenaturation, samples were loaded to gels (%5.7 stacking and %13.75 resolving) and run at a constant current of 20 mA using a Mini-PROTEAN Tetra Vertical electrophoresis-chamber and Bio-Rad Power Pac-300 (Bio-Rad, Watford, U.K.). Protein bands were visualized through Coomassie brilliant blue staining, and gel images were captured using Agfa FotoLook software (Agfa Gevaert Group, Mortsel, Belgium). Sodium caseinate (3 mg/mL) was utilized as a control for casein fragments.

Circular dichroism (CD) studies were performed to examine the changes in the secondary structures of casein fragments (α-casein (α-CN), β-casein (β-CN), and κ-casein (κ-CN)) and β-lactoglobulin (β-LG) following incubation with SiO_2_ NPs. CD measurements were conducted using a JASCO J-815 spectrometer (Easton, USA) equipped with a quartz cuvette with a 10 mm path length. Casein fragment solutions (10 mg/mL) and SiO_2_ NP solutions (21 mg/mL) were prepared in 100 mM phosphate buffer (pH 6.8). The NP-casein mixture was prepared in a 1:2 volume ratio. Spectra were recorded from 190 to 250 nm with a bandwidth of 1 nm and a scanning rate of 100 nm/min. Data analysis was conducted using CD Spectra Manager version 2. Three scans were averaged and smoothed using the Savitzky–Golay equation.^26^

The protein amounts in the corona structure of the NPs were determined using a fluorometric assay. Following the incubation of milk samples with different concentrations of SiO_2_ NPs, the samples were centrifuged at 4000*g* for 15 min. Subsequently, 150 μL of the supernatant solution was mixed with 50 μL of fluorescamine (10.8 mM) and incubated for 1 min with agitation. The fluorescence intensity of the samples was measured via Synergy H1 Hybrid Multi-Mode Microplate Reader (BioTek Instruments, Winooski, VT) at λ_ex_: 365 nm and λ_em_: 470 nm. The protein content in the corona was determined as the difference between the unbound protein found in the supernatant and the protein in the supernatant of the control samples (milk samples without NPs). All measurements were conducted in triplicate.

To investigate the change in protein hydrophobicity during interaction with Si25, we utilized ANS as a fluorescence probe following the titration approach described by Erdem.^27^ Briefly, 10× diluted milk samples were incubated with various concentrations of Si25 (1.75, 3.50, and 7.00 mg/mL) for 1 h at room temperature. Subsequently, the samples were titrated with ANS, with the ANS concentration increasing step by step from 0 to 170 μM during titration. After each titration step, the fluorescence intensity of samples was measured by Cary Eclipse fluorescence spectrophotometer (Agilent Technologies, Santa Clara, CA, U.S.A.) at λ_ex_ = 390 nm and λ_em_ = 480 nm.

### Assessment of Toxicity

Caco-2 cells were used as an *in vitro* model of the human small intestinal epithelium to assess the toxicity of ingested nanoparticles. These cells were selected due to their ability to differentiate into enterocyte-like cells, forming tight junctions, microvilli, and a functional intestinal barrier, making them suitable for studies on nanoparticle interactions and intestinal integrity assessments (TEER).^28,29^

Caco-2 cell line (An-1) was obtained from the ap Enstitüsü (Ankara, Turkey) and cultured in Dulbecco’s modified Eagle’s medium, high glucose (DMEM) medium supplemented with 10% fetal bovine serum (FBS), 100 U/mL penicillin/streptomycin, and 2.5 mM l-glutamine. Experimental procedures involved seeding cells (passages 25 to 35) into 96-well plates at a density of 5 × 10^4^ cells/well, allowing them to adhere overnight at 37 °C in a CO_2_ incubator (a humidified atmosphere of 95% air, 5% CO_2_). Caco-2 monolayers were harvested with trypsin containing 0.25% EDTA during passage and plating once a week. For treatment, the medium was substituted with a fresh cell culture medium containing digested NPs preincubated with SMUB and WM. The cells were not washed after treatment, allowing the evaluation of the viability of loosely bound and firmly attached cells.

SiO_2_ NP doses for *in vitro* toxicity analysis were determined based on the approximate daily adult SiO_2_ NP intake and the surface area of human small intestinal epithelium (2 × 10^6^ cm^2^).^30^ The working concentration of SiO_2_ was chosen as physiological dose, and it was assumed to be 4 μg mL^–1^ using referred calculations for the plate surface area (cm^2^). For high doses, 400 μg mL^–1^ (100× physiological dose) was applied in this study.

The cytotoxic effects of the NPs on Caco-2 cells were assessed through the 3-(4,5-dimethylthiazol-2-yl)-2,5-diphenyltetrazolium bromide (MTT) assay. Following NP exposure, 10 μL of MTT (5 mg/mL) was added to each well and the mixture incubated at 37 °C for 4 h. The MTT solution was discarded, and cells were solubilized in 100 μL of DMSO for 30 min with gentle shaking. Formazan was dissolved, and the solution was centrifuged (3000*g* for 1 min) to eliminate interference from SiO_2_ NPs.^31^ The absorbance was measured at 550 nm using a microplate reader (BioTek Instruments, Inc., Winooski, Vermont, USA). For control purposes, cell cultures exposed to GI fluids and digested WM samples were taken. The MTT results are shown as values relative to the control values expressed as percentages.

The total ROS level was evaluated using the nonfluorescent probe 2′,7′-dichlorofluorescin diacetate (DCFH-DA). The diacetate allows for the penetration of DCFH-DA into the cell, which is then cleaved by the cell esterase and oxidized by ROS. First, 100 μL of prewarmed Hank’s buffered salt solution (HBSS), containing 10 μM DCFH-DA, was added into each of the Caco-2 cells in 96-well plates. The cells were incubated in the dark for 30 min at 37 °C (5% CO_2_ and 95% humidity). 200 μL of radioimmunoprecipitation assay (RIPA; 50 mM Tris–HCl pH 7.4, 50 mM NaCl, 2 mM EDTA, 0.1% SDS, 1 mM DTT) buffer was added to each well after discarding DCFH-DA working solution.^32^ The plate was incubated on ice for 5 min and transferred into tubes to centrifuge (13,000*g* for 20 min) at 4 °C. The supernatant was collected into a black 96-well plate, and fluorescence intensity at λ_ex_/λ_em_: 485/530 nm was measured. Data are expressed as mean fold changes in the production compared to the negative controls.

For membrane integrity analyses, differentiated Caco-2 cell monolayer was prepared following the protocols described in the study of Lea.^33^ Briefly, 1.8 × 10^5^ cells (for maintaining the cell density ratio found in 96-well plates) from passage 28 were seeded on each transwell insert with DMEM medium and then incubated for 21 days to form a monolayer. On days 0, 7, 14, and 21, the growth of the cells and cell integrity were monitored by trans-epithelial electrical resistance (TEER) analysis. TEER analyses were conducted using the Millicell ERS-2 Electrical Resistance System (EMD Millipore Corporation, Billerica, MA, U.S.A). Analyses were performed by immersing the calibrated electrode into transwell chambers and measuring the epithelial resistance in ohms (Ω). TEER value was calculated using TEER = (*R* – *R*_0_)*A equation (*R*: resistance difference of the transwell with cell; *R*_0_: resistance difference of the transwell without cell; *A*: area of the transwell membrane). After the monolayer was formed, samples were exposed to it for 4 h. Resistance was measured at the beginning and end of the experiment. The change in the TEER value was calculated between the final and initial measurements of each transwell.

### Statistical Analyses

All data were presented as the mean ± the standard deviation (SD) from at least two independent experiments. The statistical significance was determined by using one-way analysis of variance (ANOVA) with Tukey *post hoc* test and student *t* test. *p* < 0.01 and *p* < 0.05 were considered statistically significant.

## Results and Discussion

### The Alterations in Morphology and Aggregation Behavior of NPs

XRD analysis was employed to determine the crystal structure of the SiO_2_ NPs. The resulting XRD patterns of NPs (Figure S1) revealed a characteristic broad peak at 21°, indicative of the amorphous crystal phase, consistent with previous research findings.^34^

SiO_2_ NPs underwent comprehensive physical characterization through ESEM, TEM, and DLS analyses before and after their interaction with milk samples. ESEM and TEM images demonstrated that bare NPs in SMUB have a spherical morphology (Figure 1A–C and Figure 2A,C,E). Histograms of SiO_2_ NP size were generated from TEM images (≥220 particles) using ImageJ software.^35^ The corresponding mean sizes were calculated as 20.4 ± 4.01 nm for Si25, 118.58 ± 14.20 nm for Si100, and 276.23 ± 22.92 nm for Si300 (Figure S2). The hydrodynamic diameters of Si25, Si100, and Si300 were found to be 28.9, 177.7, and 540 nm, respectively, at a particle concentration of 7 mg/mL. While bare Si300 NPs at low concentrations tended to form clusters, typically composed of two or three particles, other sizes did not exhibit this behavior (Table S1). Additionally, SEM images indicated the dispersion state of NPs, which was consistent with DLS results. Zeta potential measurements showed that bare SiO_2_ NPs were negatively charged, consistent with previous studies^36^ (Table S1).

**Figure 1:**
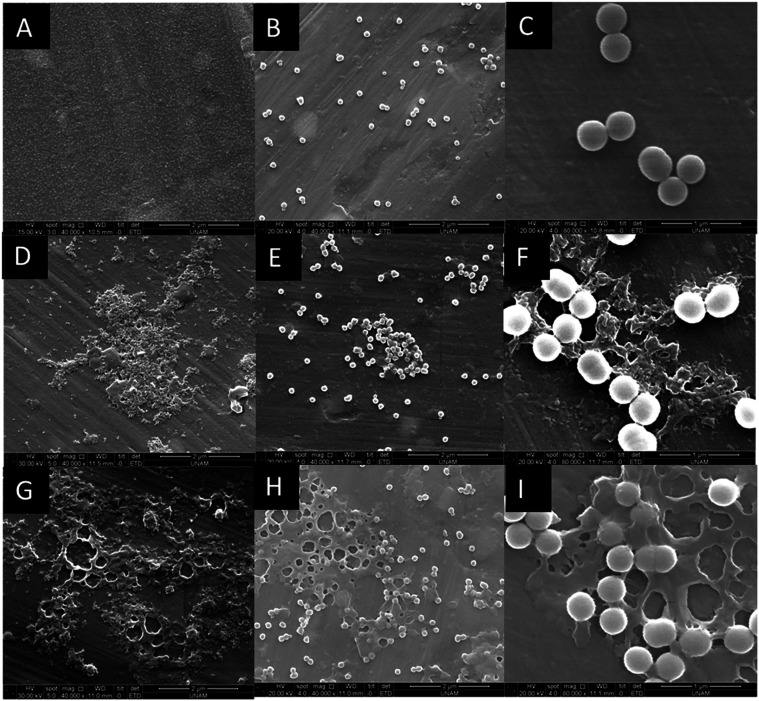
ESEM images of (A) Si25, (B) Si100, and (C) Si300 dispersed in SMUB (simulated milk ultrafiltrate buffer), (D) Si25 incubated with SM, (E) Si100 incubated with SM (skim milk), (F) Si300 incubated with SM, (G) Si25 incubated with WM (whole milk), (H) Si100 incubated with WM, and (I) Si300 incubated with WM (40,000× (A, B, D, E, G, H) and 80,000× (C, F, I) magnification).

**Figure 2:**
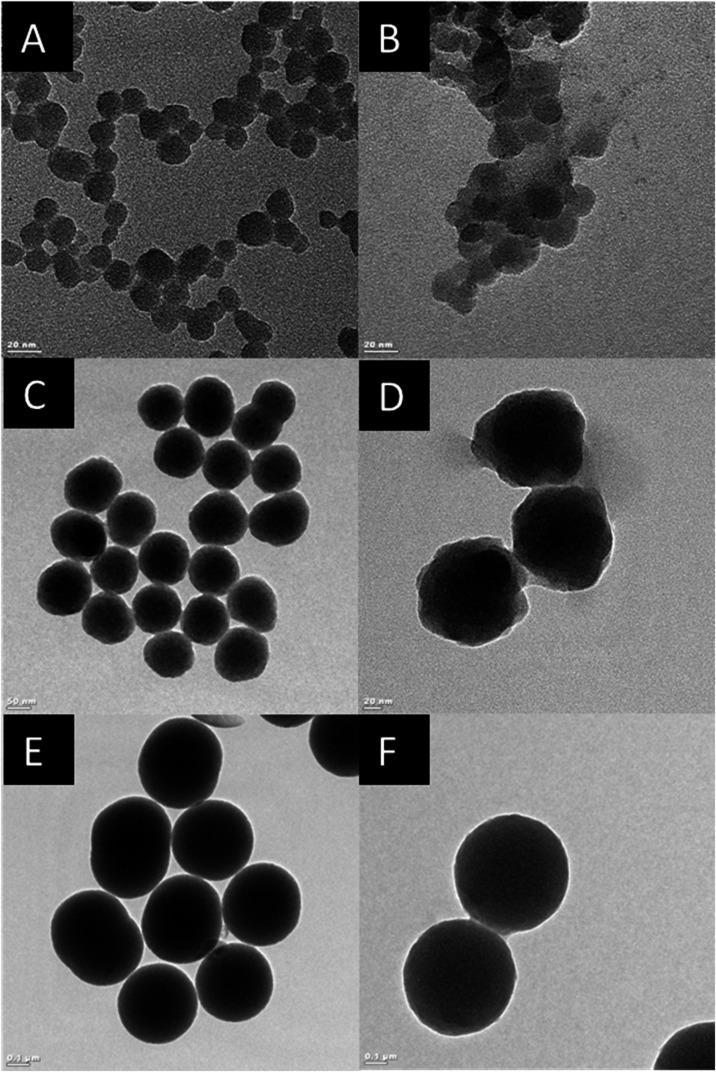
TEM images of (A) bare Si25, (B) Si25 incubated with WM, (C) bare Si100, and (D) Si100 incubated with WM, (E) bare Si300, and (F) Si300 incubated with WM.

After milk incubation, corona formation over SiO_2_ NPs was observed in all samples (Figure 2B,D,F). Corona formation after interaction between SiO_2_ NPs and different biomolecules like bovine serum albumin (BSA),^37^ lysozyme, and milk proteins^19^ has been reported previously. When Si100 and Si300 were mixed with SM and WM, their zeta potential values decreased. This reduction in zeta potential to a neutral range in milk samples is attributed to the protein adsorption onto SiO_2_ NPs.^36,38^ When Si100 and Si300 NPs were analyzed, no aggregation was observed in the ESEM images after interaction with milk samples (Figure 1E,F,H,I). DLS results indicated slightly increased hydrodynamic diameters of Si100 at all concentrations, emphasizing the formation of a protein corona (Table S1). At concentrations of 1.75 and 3.5 mg/mL, hydrodynamic diameters of Si300 decreased upon milk interaction, probably due to the dispersion of particle clusters. At a concentration of 7 mg/mL, where Si300 was already dispersed in its bare state, interaction with milk did not cause a significant change in the dispersion state and hydrodynamic diameter. This suggests that corona formation on Si300 may be less pronounced than that on particles of other sizes.

In the case of Si25, there was no significant change in the zeta potential values of particles upon interaction with milk samples. On the other hand, Si25 NPs exhibited a greater hydrodynamic diameter of 590 and 710 nm, respectively, after interacting with WM and SM (Table S1). This was further confirmed by aggregation observed in the ESEM image (Figure 1D,G) and the formation of a large particle-biomolecule cluster in the TEM image (Figure 2B). Previous studies also reported agglomeration/aggregation of small-sized SiO_2_ NP after corona formation.^39,40^ However, Yusoff, Nguyen, Chiew, Wang, and Ng^41^ demonstrated that SiO_2_ nanoparticles remained dispersed after interacting with model food ingredient solutions. Similarly, Lee, Kim, Song, Jo, Yu, Kim, Kim, Oh, and Choi^39^ reported that albumin promoted agglomeration in a SiO_2_ nanoparticle suspension, whereas the addition of glucose reduced their tendency to aggregate. In our study, the complexity of real food matrices, compared to simplified ingredient solutions, may influence the nanoparticle behavior. This variation can be attributed to differences in food components, as highlighted by McClements, Xiao, and Demokritou^20^ and Lee, Kim, Song, Jo, Yu, Kim, Kim, Oh, and Choi,^39^ which may explain the observed discrepancies.

It was observed that Si25 behaves differently from Si100 and Si300 after interacting with milk samples. Similarly, previous studies have reported that the interaction of SiO_2_ NPs with proteins is size-dependent.^42,43^ Vertegel, Siegel, and Dordick^43^ demonstrated that smaller nanoparticles primarily interact with proteins through conjugation, whereas larger nanoparticles tend to be surrounded by protein layers. Furthermore, when comparing WM and SM samples, Si25 interactions with WM result in a greater increase in hydrodynamic diameters, likely due to fat globule interference in DLS measurements.

To explore the impact of WM on the characteristics of SiO_2_ in the digestion system, an *in vitro* digestion simulation was conducted by successive incubations in SSF, SGF, and SIF. ESEM images depicting the morphological changes and hydrodynamic diameters of SiO_2_ at different stages of digestion were provided in the Supporting Information (Figures S3, S4, S5, and S6).

Both ESEM images (Figure S3) and hydrodynamic diameter measurements (Figure S6-A) indicate that bare Si25 particles maintain their dispersed state in the oral environment. Interestingly, the hydrodynamic diameters of particles preincubated with milk significantly increased in the SSF. According to Xuexin and Golding,^44^ urea may lead to a dissociation of the submicellar structure, resulting in an increase in sodium caseinate size. Therefore, the presence of urea in saliva could mitigate the hydrodynamic size of the Si25-protein corona. SGF medium caused the agglomeration of bare Si25 particles. This aligns with findings from Sohal, Cho, O’Fallon, Gaines, Demokritou, and Bello,^25^ who observed an increase in the hydrodynamic diameter of all NPs in SGF, attributed to changes in ion concentrations, sudden pH changes, and acidic characteristics of gastric fluids. For Si25 that interacted with milk, the agglomeration state was maintained in the SGF, and it was observed that their hydrodynamic diameters were larger compared to bare particles. This difference in agglomerate sizes might be due to the tendency of milk proteins adsorbed on particles to form coagulum during digestion.^45^ At the final stage of digestion, although the hydrodynamic diameters of bare particles decreased, the presence of agglomerates was observed. Postdigestion TEM images of bare particles (Figure 3A) have shown the presence of corona on the surface of the particles. NPs are unlikely to persist as bare in GI fluids, instead, they rapidly adopt a new biological and physicochemical identity by the adsorption of biomolecules on their surface.^46^ At the end of digestion, it was observed that agglomeration increased in particles preincubated with milk. Along with the increase in the particle concentration, hydrodynamic diameters also increased. Especially at 7 mg/mL particle concentration, the hydrodynamic diameter could not be measured due to large agglomerates in the medium. The large cluster structures seen before the digestion of particles interacting with milk were preserved after digestion, and the hydrodynamic diameters were significantly larger compared to bare particles.

**Figure 3:**
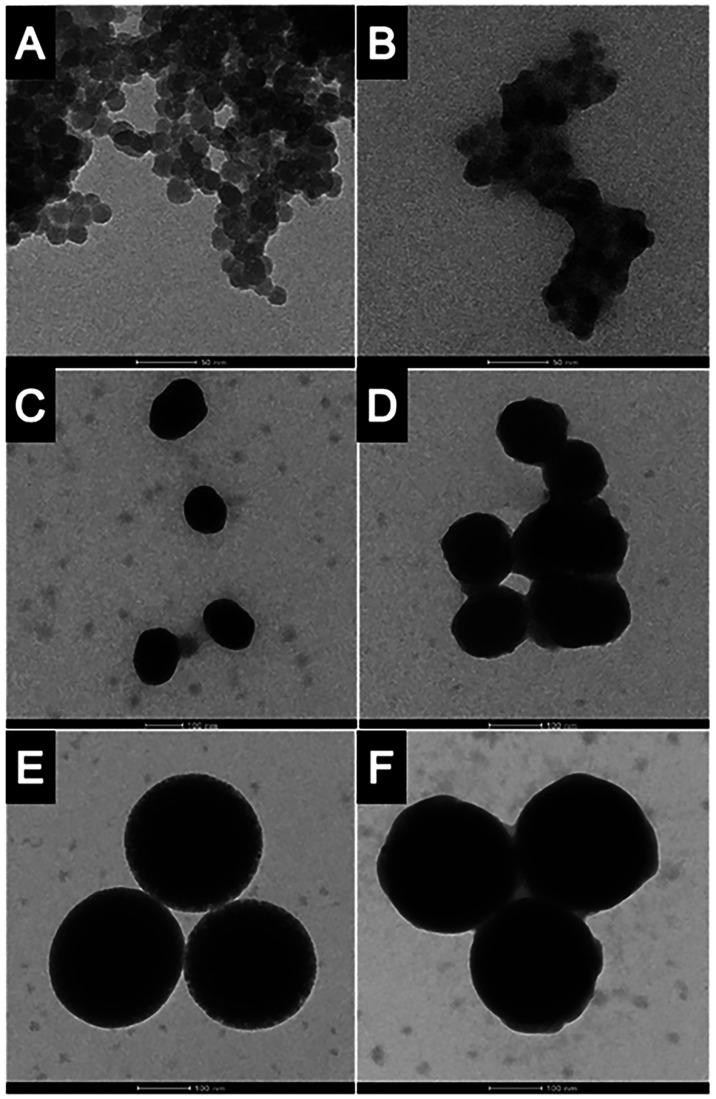
TEM images of SiO_2_ NPs after the digestion simulation. (A) Si25 preincubated with SMUB, (B) Si25 preincubated with WM, (C) Si100 preincubated with SMUB, (D) Si100 preincubated with WM, (E) Si300 preincubated with SMUB, (F) Si300 preincubated with WM.

When examining Si100 and Si300, the general trend during digestion is that the agglomerate formation, which starts in the oral phase, increased dramatically in the SGF. Upon reaching SIF, the aggregate sizes of all SiO_2_ NPs decreased due to the neutral pH values (Figures S4 and S5C,F). At the end of digestion, agglomerates were detected for both bare particles and particles preincubated with milk. Particularly at particle concentrations of 3.5 and 7 mg/mL, it was observed that the diameters of milk-incubated particles were slightly larger. TEM images (Figure 3) confirmed the presence of a corona on both bare and milk-treated particles, with a notably thicker corona in milk-exposed samples. Previous studies have investigated the behavior of nanosilica during *in vitro* digestion with and without food matrices, such as soup, coffee, and pancakes. Consistent with our findings for Si100 and Si300, nanosilica was shown to aggregate in the presence of soup and coffee after gastric digestion, followed by increased dispersion in the intestinal phase.^3^ However, contrary to our results, Peters, Kramer, Oomen, Herrera Rivera, Oegema, Tromp, Fokkink, Rietveld, and Marvin et al.^3^ reported greater dispersion of SiO_2_ nanoparticles in the intestinal phase compared to saliva. This difference may be attributed to the complex composition of milk and its relatively high protein content, which can influence nanoparticle behavior through protein corona formation.″

### Analysis of SiO_2_ Protein Corona

We examined the formation of a protein corona on SiO_2_ NPs with varying sizes and concentrations after their interaction with WM and SM. To assess the protein amount on the NP surfaces, we employed the fluorescamine assay. Figure 4 illustrates the protein amount (%) present in milk samples that were adsorbed onto the NPs. As anticipated, a higher concentration of SiO_2_ NPs resulted in a more pronounced protein corona, with the highest bound protein ratio observed in both SM and WM samples at 7 mg/mL of Si25. The amount of protein in protein corona over NPs generally depends on their size.^47,48^ Si25 has a higher surface area than Si100 and S300 at the same concentration, increasing the rate of protein adsorption.^49^ Also, Si25 has a thicker protein layer than Si100 and 300 after interaction with WM in TEM images (Figure 2B,D,F). However, no significant difference in the amount of bound protein was observed between Si100 and Si300. Considering the similar aggregation states of NPs (Figures 1 and 2), Si100 and Si300 may exhibit similar protein corona formation when exposed to milk samples. Also, no significant differences were observed between the amounts of adhered protein in the WM and SM samples.

**Figure 4:**
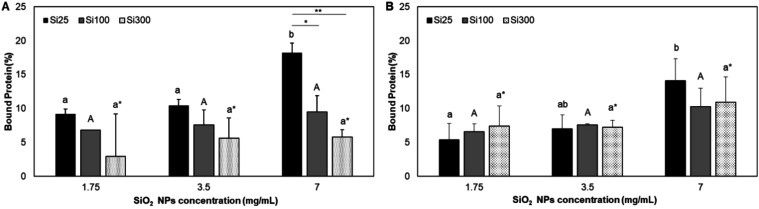
Percentage of milk proteins adsorbed on NPs in (A) WM and (B) SM samples obtained from fluorescamine assay. Different letters indicate the significant difference between samples that have the same size, at *p* < 0.05; * indicates the significant difference between different particle sizes at the same concentration *: *p* < 0.05, **: *p* < 0.01.

To determine the protein composition of the corona structure on NPs that were incubated with WM and SM, SDS-PAGE analyses were performed (Figure S7). The composition of protein corona for all sizes, α- and β-caseins, was observed to be more abundant compared to whey proteins with respect to their relative abundance in the milk. Also, large proteins tend to interact with NPs with greater affinity than small proteins, which is significant in the context of protein corona formation in complex biological media where large protein complexes are present.^50^ Consistent with the fluorescamine data (Figure 4), Si25 adsorbed more proteins than other diameters, which resulted in a higher protein band intensity in (Figure S7). It seems likely that particle size affects the protein corona quantitatively rather than qualitatively, as the same protein bands are observed in the gel.^48^

Weakly bound proteins, known as the *soft corona*, could be lost during separation steps, while tightly bound proteins, called the *hard corona*, remain adsorbed.^51^ In this study, we investigated the hard corona layer of the SiO_2_ NPs. As shown in Figure S7, SiO_2_ NPs can bind α-CN, β-CN, β-Lg, and α-lactalbumin (α-La).

Following incubation with SiO_2_ NPs, secondary structural alterations in casein fragments and β-lactoglobulin were analyzed using CD spectroscopy. The obtained CD spectra are presented in Figure S8. Secondary structural components, including α-helix, β-strand, β-turn, and random coil, were quantified using BeStSel online software.^52^ As shown in Table S2, the random coil structure was the predominant element in α-, β-, and κ-caseins. The CD values for α- and β-caseins correspond with prior literature findings.^53^ The alterations in the secondary structure were predominantly noted in casein fragments, particularly the α- and β-subunits (Figure S8). Previous studies have demonstrated that nanoparticle–protein interactions can induce conformational changes in proteins such as BSA,^54^ HSA,^55^ lysozyme,^43^ and α-casein.^56^ Si25 affected the secondary structure to a lower extent and in a different manner from Si100 and Si300. A decrease in helicity and an increase in disorder were observed in α-casein following interaction with Si100 and Si300, whereas Si25 led to a slight increase in the helical content. In β-casein, an increase in the helical content was observed with all nanoparticle sizes. However, Si100 and Si300 caused a reduction in the β-sheet structure, while Si25 induced an increase.

Physical properties such as size, shape, and surface area influence protein structural changes upon nanoparticle binding.^57^ The size-dependent effects suggest that smaller SiO_2_ NPs may have different binding affinities and structural impacts in comparison to larger ones. These variations in conformational changes are likely due to differences in nanoparticle surface curvature.^42,43,58^

The literature demonstrates that SiO_2_ NPs can interact with proteins via hydrophobic forces,^59,60^ hydrogen bonds, and electrostatic interactions.^59^ Based on electrostatic interactions, previous studies revealed that negatively charged particles would primarily attract positively charged proteins.^61^ However, in the case of milk proteins, negatively charged SiO_2_ NPs also interacted with casein fragments displaying negative charge at neutral pH values.^62^ Similar results have been reported for interactions between SiO_2_ NPs and negatively charged proteins.^48,63^ Therefore, the interaction between casein and NPs may be influenced by the uneven charge distribution of casein fragments, which allows for electrostatic interactions.^62,64^ However, compared to electrostatic attraction/repulsion, other driving factors, such as hydrophobic interactions, appear to play a more significant role in the silica-corona interactions.^48,59,60,63^ We propose that the interaction between SiO_2_ NPs and casein primarily occurs through the hydrophobic regions of casein micelles given the hydrophobicity of α-CN and β-CN.

To reveal the presence of a hydrophobic interaction, we investigated the change in the surface hydrophobicity of milk proteins during interaction with Si25. For this purpose, we conducted ANS assessments. ANS, known for its binding ability to hydrophobic regions of proteins, was employed as a tool for probing protein binding.^27^ ANS titration curves of milk proteins interacted with and without Si25 are provided in Figure S9. The decrease in fluorescence intensity at the highest ANS concentration observed during the interaction of Si25 with milk proteins suggests a reduction in the surface hydrophobicity of milk proteins. This decrease may be attributed to the hydrophobic interaction between Si25 and milk proteins, owing to their hydrophobic nature.^65^ Another reason could be that the interaction between Si25 and milk proteins leads to steric hindrance, potentially masking the hydrophobic surfaces. Besides hydrophobic interaction, hydrogen and Si–O–Ca bonds may also play an important role in the interaction between SiO_2_ and milk proteins. Previous research done by He, Zeng, Liang, Long, and Xu^66^ suggested that Si could substitute for Ca^2+^ in the calcium phosphate and form Si–O–Ca bonds. Additionally, silanol groups on SiO_2_ NPs are capable of forming hydrogen bonds with water molecules.^67^ Nevertheless, further research is necessary to explore the characteristics of the interaction between Si25 and milk proteins.

Furthermore, we investigated the change of protein profile during *in vitro* digestion simulation in the absence and presence of WM. Although a thin corona layer was observed in the TEM images of bare NPs after digestion, no protein bands were detected in the SDS-PAGE analysis of all digestive fluids, probably due to the low sensitivity of SDS-PAGE. Similarly, in the research using magnetic silica nanoparticles with skim milk solution, tofu, and peanut, no food protein band was determined at the end of the digestion stage.^68^ In the presence of WM, for all sizes of SiO_2_ NPs, we observed more intense α-casein bands in saliva compared to nondigested NPs (Figure S10A–C, line 2). Surprisingly, when WM was present, Si25 NPs in SGF revealed the presence of pepsin in the protein corona structure (Figure S10A, line 3). However, the pepsin bands did not appear in the absence of WM (Figure S10A, line 4). This can be attributed to the self-digestion of pepsin in the solution without other proteins. In SGF, milk proteins, except for the β-Lg, disappeared for all sizes of NPs (Figure S10, lines 3, 10, 15, and 24) due to the.^68,69^ It has been suggested that lipophilic molecules, such as fatty acids and triglycerides, bind to β-L.^70,71^ Additionally, we observed dense bands at the bottom of the gel, indicating peptide residues of protein corona of Si25, Si100, and Si300 in SGF (Figure S10, A lines 3–4, B, and C lines 4–5). Although the corona structure of all sizes of NPs was observed in SIF in the ESEM results (Figures S3, S4, and S5).^68^ For further studies, more sensitive methods should be used to determine the peptide profile of the protein corona structure of NPs after digestion.

### Nanoparticle Toxicity

It is well known that the medium in which NPs are located determines all their physicochemical properties, which can result in alterations in zeta potential, aggregation state, cytotoxicity, and cellular uptake.^28,72^ Here, we used digested SiO_2_ NPs and the colon epithelial cell line Caco-2 as a model to investigate the biological impact of **nanoparticle size** and the **presence of food matrix**. Figures 5 and 6 show the cytotoxicity of different sizes of SiO_2_ NPs in the presence and absence of WM.

**Figure 5:**
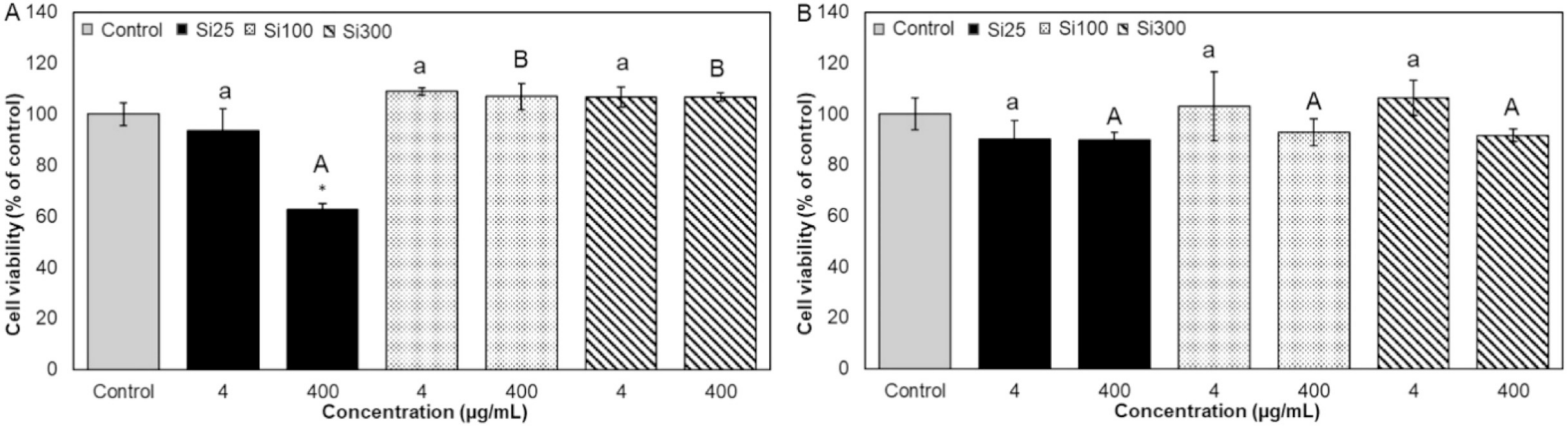
Effect of digested SiO_2_ NPs (preincubated in SMUB (A) and WM (B)) on Caco-2 cell viability. The viability of Caco-2 cells was probed with the MTT assay. Results were expressed as a percentage (%) of the control cells (exposed to digestion fluid and digested WM). It was presented as average ± standard deviation, *n* ≥ 3; * indicates significant differences between sample and control. At *p* < 0.01. Different letters indicate significant differences between samples at different sizes *p* < 0.01.

**Figure 6:**
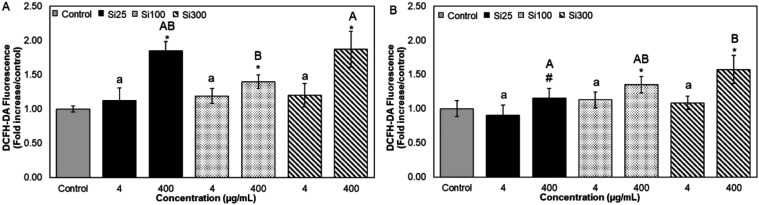
Effect of digested SiO_2_ NPs (preincubated in SMUB (A) and WM (B)) on total ROS generation in Caco-2 cells. ROS generation was measured by DCFH-DA fluorescence. Results were expressed as fold increase over controls. It was presented as average ± standard deviation, *n* ≥ 3; * indicates that significant differences between samples and controls at *p* < 0.01; # indicates significant differences between NPs in WM and SMUB samples at *p* < 0.01. Different letters indicate significant differences between different particle sizes at the same concentration at *p* < 0.01.

Cell viability of approximately 60% was observed after treatment with digested bare Si25 at 400 μg/mL, while other sizes did not show any significant alteration (Figure 5A). Following digestion simulation, NPs are assumed to have a thin corona layer from digestion fluids, and the serum in the cell culture medium inevitably binds and forms a protein corona around them before cell exposure.^73^ The aggregation state and thin corona layer did not prevent the cytotoxicity of Si25. To compare the effect of digestion simulation on bare NP behavior, we found that undigested bare Si25 showed the same cell viability percentage (∼65%, data not shown) as that of digested Si25, indicating that the digestion process did not affect cell viability. McCracken, Zane, Knight, Dutta, and Waldman^74^ suggested that while TiO_2_ NPs reduce cell viability after digestion because of the toxic components of protein corona on the TiO_2_ surface, ingested SiO_2_ NPs did not induce cell apoptosis under the same conditions. In our case, the similarity in behavior between digested and undigested bare Si25 is likely due to the presence of comparable corona layers and aggregation states. During exposure in the MTT assay, we can speculate that digested and undigested Si25 NPs, all particles were suspended in DMEM in the working well, which means that particles most certainly had similar corona layers. Consistent with this, the literature reports the aggregation of 30 nm SiO_2_ in DMEM to be similar in aggregation behavior to our results for Si25 after digestion.^30^

Contrary to Si25, bare Si100 and bare Si300 did not inhibit cell viability. The relationship between NP size and cytotoxicity is well documented, generally indicating that smaller particles are more toxic due to their increased surface area, which enhances their interaction with biological systems.^36,75,76^ Napierska, Thomassen, Rabolli, Lison, Gonzalez, Kirsch-Volders, Martens, and Hoet^75^ previously reported that the cytotoxicity of monodisperse amorphous silica particles was strongly related to particle size. Similarly, Wottrich, Diabaté, and Krug^77^ found dose-dependent effects in cells treated with 60 nm SiO_2_ NPs, while the 100 nm particles showed no such response, with smaller particles showing significantly greater cytotoxicity than larger ones.^78^

In the presence of WM, for all sizes of NPs, there were no significant alterations in cell viability at both 4 and 400 μg/mL concentrations (*p* < 0.05) (Figure 5). The formation of the protein-Si25 corona may appear to mitigate toxicity by preventing the NPs from reaching the cell surface. Additionally, variations in the compositions of the protein coronas between digested Si25 and digested whole WM-incubated Si25 could lead to different toxicity profiles due to potential differences in the cellular response.^79^ Notably, the presence of WM led to the formation of large aggregates of digested Si25 (∼5 μm), reducing their toxicological relevance^80^ and highlighting the crucial role of aggregate size in determining their toxicity. In our study, the presence of the food matrix did not affect the aggregate sizes of other particles (Si100 and Si300) after digestion and did not change the toxicity of the NPs.

The aggregation state of NPs can have a dual effect, either mitigating^81^ or inducing/increasing^82^ cytotoxicity. In this context, previous studies have demonstrated that the aggregation state of SiO_2_ nanoparticles can influence their toxicity. For instance, Murugadoss, van den Brule, Brassinne, Sebaihi, Mejia, Lucas, Petry, Godderis, Mast, and Lison et al.^83^ found that aggregation reduced the toxicity of SiO_2_ nanoparticles in Caco-2 cells. Conversely, another study reported that synthetic amorphous silica nanomaterials with larger aggregate sizes (∼2.5 μm) significantly impacted cell proliferation.^84^ However, it is essential to note that these studies utilized pristine nanoparticles and did not take into account the effects of protein corona formation and *in vitro* digestion on toxicity. In our case, we speculated that the formation of the corona-Si25 complex had a more significant influence on toxicity compared to aggregation. Further research is warranted, particularly a qualitative analysis of the protein corona composition, as this is crucial for understanding the mechanisms behind the toxicity of nanoparticles. In evaluating the cytotoxicity of nanoparticles, it is essential to integrate alternative assays alongside the MTT assay to enhance the robustness of our findings. In future studies, incorporating methods such as the WST-8 assay can provide a more comprehensive understanding of cell viability by capturing different aspects of cellular responses.

Numerous studies have demonstrated the inhibitory effect of the protein corona on NP cytotoxicity. For instance, Docter, Bantz, Westmeier, Galla, Wang, Kirkpatrick, Nielsen, Maskos, and Stauber^72^ reported that exposure to 30 nm SiO_2_ NPs significantly reduced cell viability; however, this effect was mitigated upon the formation of a protein corona. Also, corona formation decreased the cellular uptake of 30 nm SiO_2_ NPs. In another study, 50 nm silica NPs lead to cell damage in the absence of serum, but this damage is mitigated in the presence of serum due to the adhesion properties of the NPs under different conditions.^85^ The probable cause for this reduction in toxicity is the decreased surface energy of the bare SiO_2_ due to the formation of a protein corona. The toxicity of SiO_2_ NPs is linked to the presence of surface silanol (≡SiOH) groups,^86,87^ which can interact with membrane components through hydrogen bonding. The cumulative interaction between surface silanol groups and phospholipids can cause cell membrane lysis.^36,87^ Amorphous silica contains a notable amount of strained 3-membered siloxane rings, which generate surface-associated radicals. These radicals react with water to produce highly reactive hydroxyl radical HO^•^ that can interact with cells, initiating inflammatory responses and potentially causing cell death. Since surface silanol groups of SiO_2_ are covered by proteins, it is not surprising that this phenomenon inhibits the adhesion of SiO_2_ NPs to the cell surface.^36^ Thus, the protein corona can block the particle surface and reduce direct contact of NPs with cell membranes, thereby protecting cells from membrane disruption and acute cytotoxicity.^88^

Cell barrier integrity is important for the transport and absorption of NPs. Following the cell viability results, we performed TEER analysis for a 4 h period to investigate the impact of digested Si25 NPs on cell integrity. Notably, at low concentrations, both bare Si25 and WM-incubated Si25 did not cause significant damage to the cell integrity (Figure S11). However, when exposed to high concentrations of NPs, the monolayer was affected, resulting in a significant decrease in the TEER value. Bare Si25 caused a more dramatic decrease in epithelial resistance than WM-Si25 did (Figure S11). The literature also approves this result which indicates that the presence of proteins decreases the bare NPs’ association with cells and tight junctions due to the formation of a biomolecular corona.^89^

The toxicity of NPs is closely linked to their ability to trigger the production of ROS.^90^ To investigate this relationship, we exposed cells to digested bare SiO_2_ NPs to evaluate the ROS production levels. Our results showed that at low concentrations, Si25, Si100, and Si300 did not induce a significant increase in ROS production. However, at a concentration of 400 μg/mL, we observed a marked elevation in ROS generation with fold changes of 1.85, 1.40, and 1.87, respectively (Figure 6A). Consistent with previous studies, SiO_2_ NPs have been shown to induce ROS.^76,91^ SiO_2_ NPs can generate ROS through surface catalytic reactions.^92^ Furthermore, interactions of SiO_2_ NPs with the cells may disrupt cellular membrane integrity, leading to lipid peroxide production and subsequent ROS generation.^93^ Additionally, the induction of mitochondrial dysfunction or cellular response by SiO_2_ NPs may induce ROS production.^94^ Although Si100 and Si300 did not cause a loss of cell viability, we observed considerable ROS generation after digestion. This suggests that Si100 and Si300 might interact with cells in a way that does not lead to significant cytotoxicity despite inducing ROS production. This phenomenon has been reported previously for other nanoparticles.^31^

The observation that SiO_2_ NPs induce ROS production without causing significant cell viability loss can be attributed to several factors. While ROS production is often associated with cell toxicity, the levels of ROS generated by the SiO_2_ NPs might not be sufficient to induce cell death.^95^ Additionally, the exposure time to SiO_2_ NPs might be too short to observe a significant cell viability loss. ROS production can be an early event, and cell viability loss might occur at later time points.^28^ Another explanation for the observed ROS production without significant cell viability loss might be the activation of the cellular defense mechanism, such as antioxidant enzymes.^96^ Previous studies have suggested that cells may counteract oxidative stress through enzymatic responses, including catalase, glutathione, or superoxide dismutase activity.^31,95^ However, we acknowledge that our study did not directly measure oxidative stress markers, and further investigations are required to confirm whether such mechanisms contribute to the observed toxicity effects. The complexity of NP-cell interactions must be investigated further to elucidate the mechanisms underlying NP-induced ROS production and cytotoxicity.

Interestingly, while Si25 did not increase ROS generation, Si100 and Si300 led to a notable alteration in ROS generation in the presence of WM significantly (Figure 6B). This difference in ROS generation could be attributed to the formation of a protein corona on the surface of the NPs, which can modulate their interactions with the cells. As previously reported, a protein corona may decrease NP adhesion to the cell membrane by reducing the surface-free energy after binding with proteins, thus decreasing particle–cell interactions.^97^ Moreover, the protein corona could modulate the surface charge, hydrophobicity, or reactivity of NPs, which in turn could influence their cellular uptake and intracellular behavior.^98^ In the case of Si25, WM incubation significantly mitigated ROS generation compared to the no-WM condition (*p* < 0.05). This reduction could be related to the formation of a unique Si25-protein corona and aggregation behavior of Si25 after WM digestion, which differs from Si100 and Si300. It also led to significant differences between Si25 and Si300 in the presence of WM (Figure 6B). The distinct protein corona structure and aggregation behavior of Si25 may have contributed to its reduced ROS generation in WM-incubated conditions. In contrast, Si100 and Si300 induced ROS production in WM-incubated conditions, but there was no statistically significant difference between digested bare and digested WM-incubated conditions in ROS generation at 400 μg/mL. This suggests that Si100 and Si300 may have similar corona structures, which could have contributed to their similar ROS generation profiles in milk-incubated conditions. The trends in the results indicate that digested WM-incubated SiO_2_ NP concentrations did not cause significant cell death. However, we observed a trend of elevated ROS generation, suggesting that while direct toxicity was not evident, there is a potential for subtoxic effects that could lead to toxicity *in vivo*.^28^

Altogether, these results provide more insights into *in vitro* digestion on NPs in WM and thereby contribute to the assessment of the potential health impact of NPs in food. However, our results should be interpreted with caution due to the limitations of the Caco-2 cell model. Specifically, our experiments were conducted in undifferentiated Caco-2 cells, which may not fully reflect the in vivo situation. Nevertheless, this model can be regarded as a screening tool for identifying potential hazards before undertaking further investigation using differentiated cells and coculture models.^76^ Our findings have implications for the safe use of SiO_2_ NPs in consumer products and highlight the need for further research into the mechanisms underlying NP-induced ROS production and cytotoxicity.

## Conclusions

In conclusion, this study pioneers the investigation of SiO_2_ NPs behavior in real food matrices, specifically skim and whole milk, during *in vitro* digestion. We demonstrate that the interaction of SiO_2_ NPs with milk leads to the formation of a protein corona, which affects the NP agglomeration and aggregation behavior. The size of the NPs was found to influence this behavior, with smaller NPs (Si25) exhibiting aggregation, while larger NPs (Si100 and Si300) showed decreased zeta potential values indicative of protein adsorption without aggregation. The presence of milk proteins, particularly α- and β-caseins, was found to play a significant role in the protein corona structure, and hydrophobic interactions were identified as a key factor in silica-corona interactions. Our *in vitro* digestion simulation revealed that the behavior of SiO_2_ NPs changes significantly throughout the digestive process, with agglomerate formation increasing in the oral phase and dramatically in the gastric phase, followed by a decrease in aggregate sizes in the intestinal phase. However, Si25 differed, having the aggregation to a larger hydrodynamic size after the intestinal phase because of the Si25-protein corona.

To the best of our knowledge, previous studies have not considered the effects of the size, real food matrix, and digestion together on nanoparticle toxicity. Our study addresses this gap by offering a more realistic scenario. The size of the NPs was found to play a critical role, with smaller NPs (Si25) exhibiting cytotoxicity and larger NPs (Si100 and Si300) showing no cytotoxic effects. While these sizes were selected to simulate the distribution of nanoparticles, further studies including a broader range of sizes would be useful to better characterize the size-dependent toxicity of SiO_2_ NPs. On the other hand, the presence of milk mitigated the cytotoxicity of Si25 NPs, likely due to the formation of a protein corona that reduced NP-cell interactions. However, having a protein corona layer did not significantly decrease the ROS generation of Si100 and Si300, while the Si25-protein corona reduced ROS production. It is clear that the aggregation state and the way corona formation occurs play important roles in NP toxicity. Overall, our study highlights the importance of considering the effects of the food matrix and digestion on NP behavior and toxicity. It suggests that the unique NP-protein corona and aggregation behavior of Si25 NPs may contribute to its reduced ROS generation and cytotoxicity under milk-incubated conditions. While our findings suggest potential biological effects of Si25 on intestinal cells, further studies (e.g., with complex cocultures or *in vivo* models) would be valuable to fully understand the mechanisms of the impact of protein corona on NP-cell interactions.

The bioavailability and cellular uptake of SiO_2_ NPs depend on their aggregation state and protein corona formation, which would affect interaction with intestinal barriers. Food matrix interaction following digestion may alter NP uptake and translocation. Further studies are needed to clarify their systemic exposure and long-term effects. These future investigations will be crucial to enhancing our understanding of the potential health implications of SiO_2_ nanoparticles in food.

On the other hand, EFSA has highlighted the need for further research to address uncertainties regarding the potential toxicological effects of nanosized aggregates in silicon dioxide (E 551), particularly concerning their characterization and interactions with biological systems. Our study has important implications for food safety regulations and nanoparticle risk assessment, as it highlights the need to consider the effects of food matrices and digestion on NP behavior and toxicity. The observed size-dependent cytotoxicity and protective role of the protein corona emphasize the necessity of integrating realistic exposure conditions into regulatory frameworks to better assess the safety of nanomaterials in food products.

## Supplementary Material



## Notes

Hydrodynamic diameter, polydispersity index (PDI), and ζ-potential values of SiO_2_ NPs (Si25, Si100, and Si300) in skim milk (SM) and whole milk (WM); XRD patterns of Si25, Si100, and Si300, size distributions of SiO_2_ NPs; SEM images of digested bare SiO_2_ NPs and digested SiO_2_ NPs preincubated with WM; hydrodynamic diameters of SiO_2_ NPs in different digestion fluids; SDS-PAGE profiles of SiO_2_ NPs interacted with milk samples; CD spectra and secondary structure ratio of α-, β-, κ-casein, and β-Lg before and after interaction with milk samples; ANS titration curves of WM and SM in the absence and presence of Si25; SDS-PAGE profiles of SiO_2_ NPs through the digestive system; and TEER change during the monolayer formation process of Caco-2 cells treated with WM-incubated NPs and nonincubated Si25 (PDF)

## Abbreviations

NP: nanoparticle
Si25: 25 nm SiO_2_ nanoparticle
Si100: 100 nm SiO_2_ nanoparticle
Si300: 300 nm SiO_2_ nanoparticle
WM: whole milk
SM: skim milk
SMUB: simulated milk ultrafiltrate buffer
SSF: simulated saliva fluid
SGF: simulated gastric fluid
SIF: simulated intestinal fluid
