# Supplementary material for: Impact of Milk on the Behavior and Toxicity of Nanosized SiO2 Particles during In Vitro Digestion Simulation
Source: ACS Omega. 2025 Jun 12;10(24):26148–63. doi: 10.1021/acsomega.5c03484 (PMC12198997; doi:10.1021/acsomega.5c03484)
Supplement: Supplementary file 1 [file ao5c03484_si_001.pdf]

Impact of Milk on the Behavior and Toxicity of Nano-Sized SiO_2 Particles During *in vitro* Digestion Simulation

SUPPLEMENTARY DATA

Hafize Öz Elrai[1], Nazım Sergen Mısırlı[1,2], Esin Bayan Karakadılar[1,2], Seda Yildirim-Elikoğlu[2], Fahriye Ceyda Dudak[2*]

[1]*Graduate School of Science and Engineering, Hacettepe University, Beytepe 06800, Ankara, Turkey*

[2]*Department of Food Engineering, Hacettepe University, Beytepe 06800, Ankara, Turkey*

*corresponding author: Fahriye Ceyda Dudak

e-mail address: ceyda@hacettepe.edu.tr

Table S1. Hydrodynamic diameter, polydispersity index (PDI) and ζ-potential values of SiO_2 NPs in skim milk (SM) and whole milk (WM).

Diameter	Concentration (mg/mL)	Sample	PDI	Hydrodynamic diameter	ζ-potential (mV)
	Milk samples	WM	0.375	1144.2	-
		SM	0.288	500.7	-
25 nm	1.75	SiO_2	0.275	28.8 ± 1.1	-13.52 ± 0.53
		SiO_2 +WM	0.280	697.8 ± 139.9	-12.42 ± 1.06
		SiO_2 +SM	0.271	422.0 ± 148.2	-12.00 ± 0.99
	3.5	SiO_2	0.226	30.9 ± 5.1	-11.38 ± 0.71
		SiO_2 +WM	0.240	571.8 ± 51.0	-11.65 ± 1.25
		SiO_2 +SM	0.247	524.4 ± 61.8	-11.70 ± 0.87
	7.0	SiO_2	0.271	28.7 ± 1.8	-10.90 ± 1.28
		SiO_2 +WM	0.247	706.8 ± 59.3	-11.79 ± 1.75
		SiO_2 +SM	0.263	587.7 ± 13.5	-11.83 ± 0.90
100 nm	1.75	SiO_2	0.669	168.6 ± 0.7	-24.33 ± 1.12
		SiO_2 +WM	0.459	344.9 ± 25.6	-10.20 ± 0.84
		SiO_2 +SM	0.249	312.0 ± 6.4	-9.88 ± 1.04
	3.5	SiO_2	0.160	195.5 ± 3.0	-22.00 ± 1.43
		SiO_2 +WM	0.349	298.7 ± 31.4	-10.50 ± 0.60
		SiO_2 +SM	0.192	261.0 ± 20.2	-9.69 ± 1.25
	7.0	SiO_2	0.113	177.7 ± 6.2	-25.70 ± 0.93
		SiO_2 +WM	0.218	259.5 ± 23.55	-11.58 ± 0.66
		SiO_2 +SM	0.114	238.3 ± 15.1	-13.44 ± 2.28
300 nm	1.75	SiO_2	0.458	1127.0 ± 559.4	-18.15 ± 1.01
		SiO_2 +WM	0.238	597.0 ± 95.95	-11.1 ± 1.22
		SiO_2 +SM	0.217	540.4	-12.2 ± 1.13
	3.5	SiO_2	0.442	1213.0 ± 496.0	-20.30 ± 0.69
		SiO_2 +WM	0.223	619.3 ± 46.67	-11.82 ± 0.98
		SiO_2 +SM	0.133	517.0 ± 29.6	-12.70 ± 2.07
	7.0	SiO_2	0.097	540.8 ± 21.5	-20.7 ± 1.35
		SiO_2 +WM	0.199	547.6 ± 49.92	-11.97 ± 1.47
		SiO_2 +SM	0.093	497.4 ± 22.8	-12.45 ± 1.67

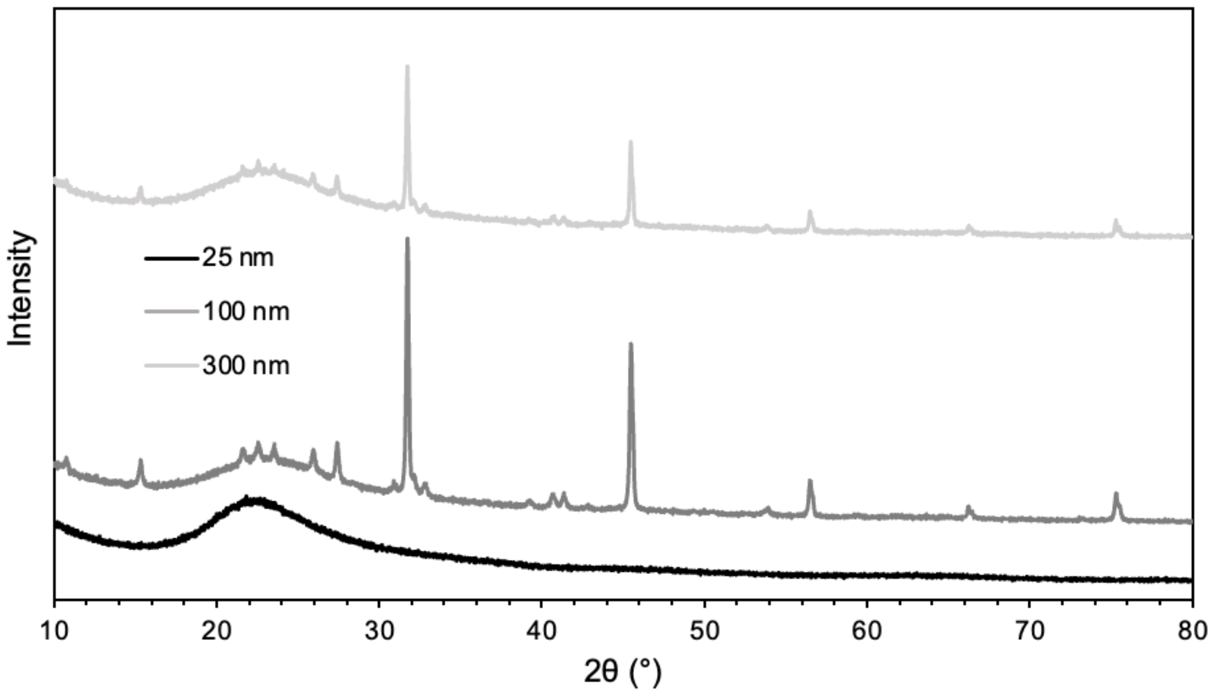

Figure S1. XRD patterns of Si25, Si100 and Si300. The peaks at 27.4°, 31.7°, 45.4°, 56.4° and 66.2° of 100 and 300 nm SiO2 samples that obtained by drying NPs in SMUB, belong to sodium chloride [1]

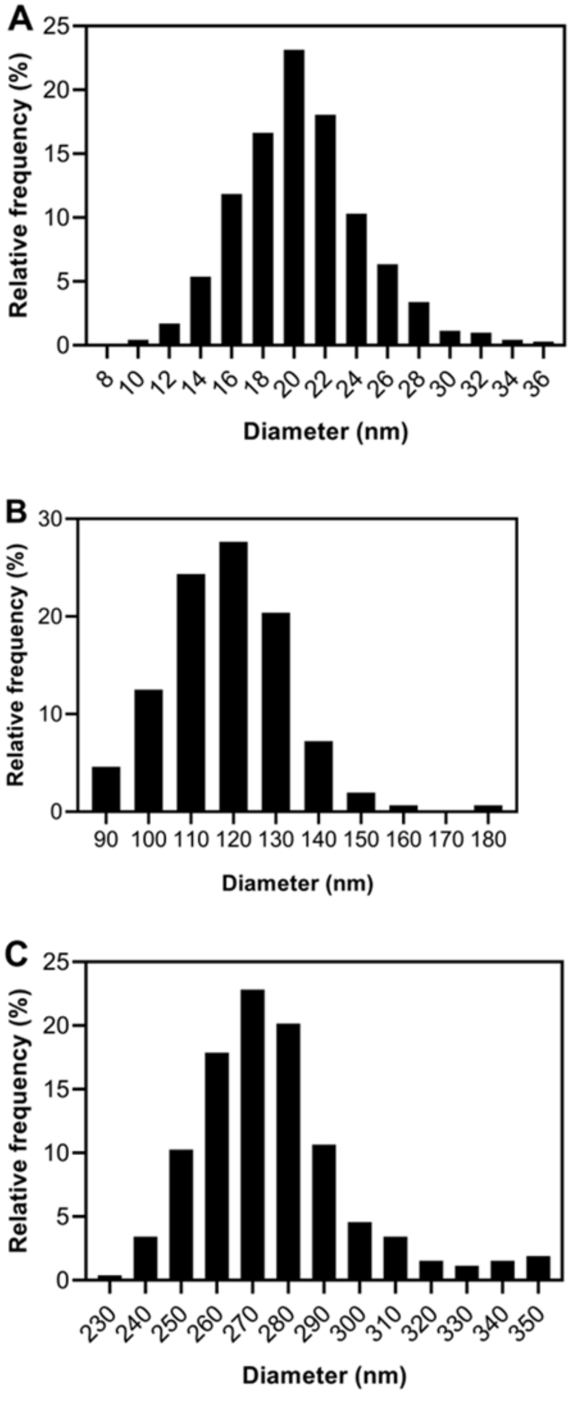

Figure S2. Size distributions of A) Si25, B) Si100 and C) Si300.

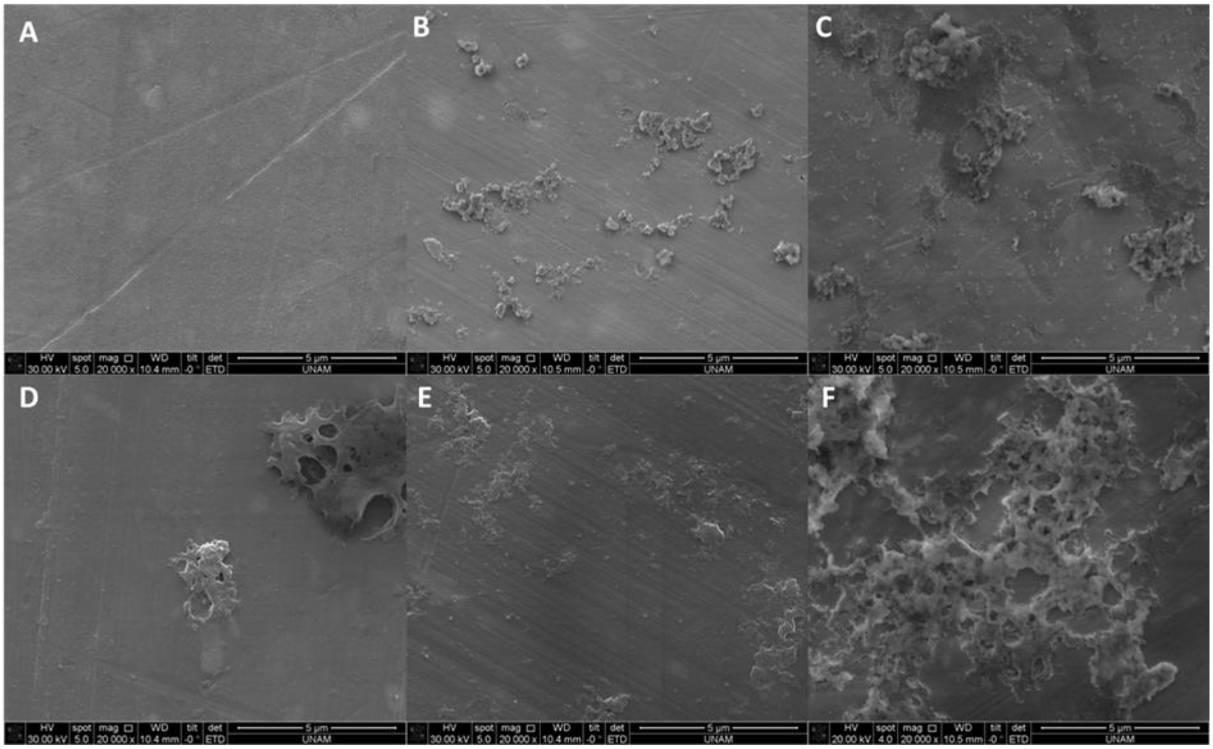

Figure S3. SEM image of digested bare Si25 NPs in A) SSF, (B) SGF, C) SIF and digested Si25 NPs pre-incubated with WM in D) SSF, E) SGF, F) SIF.

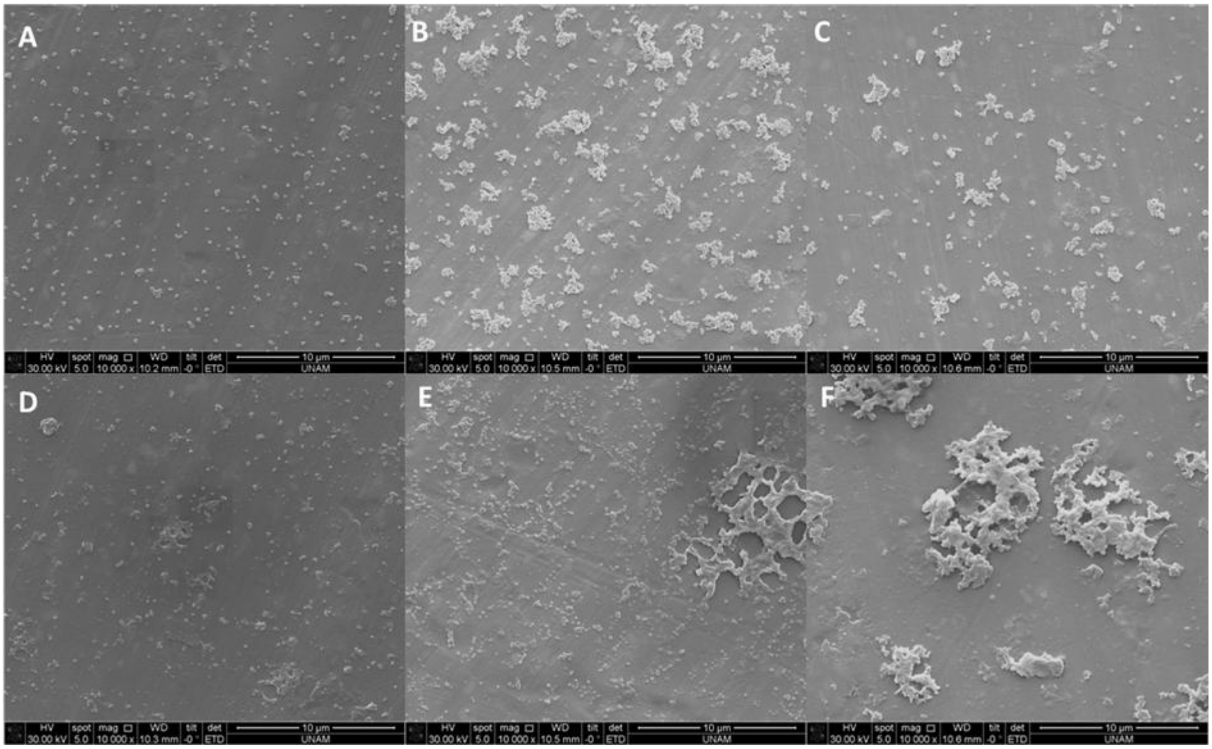

Figure S4. SEM image of digested bare Si100 NPs in A) SSF, (B) SGF, C) SIF and digested Si100 NPs pre-incubated with WM in D) SSF, E) SGF, F) SIF.

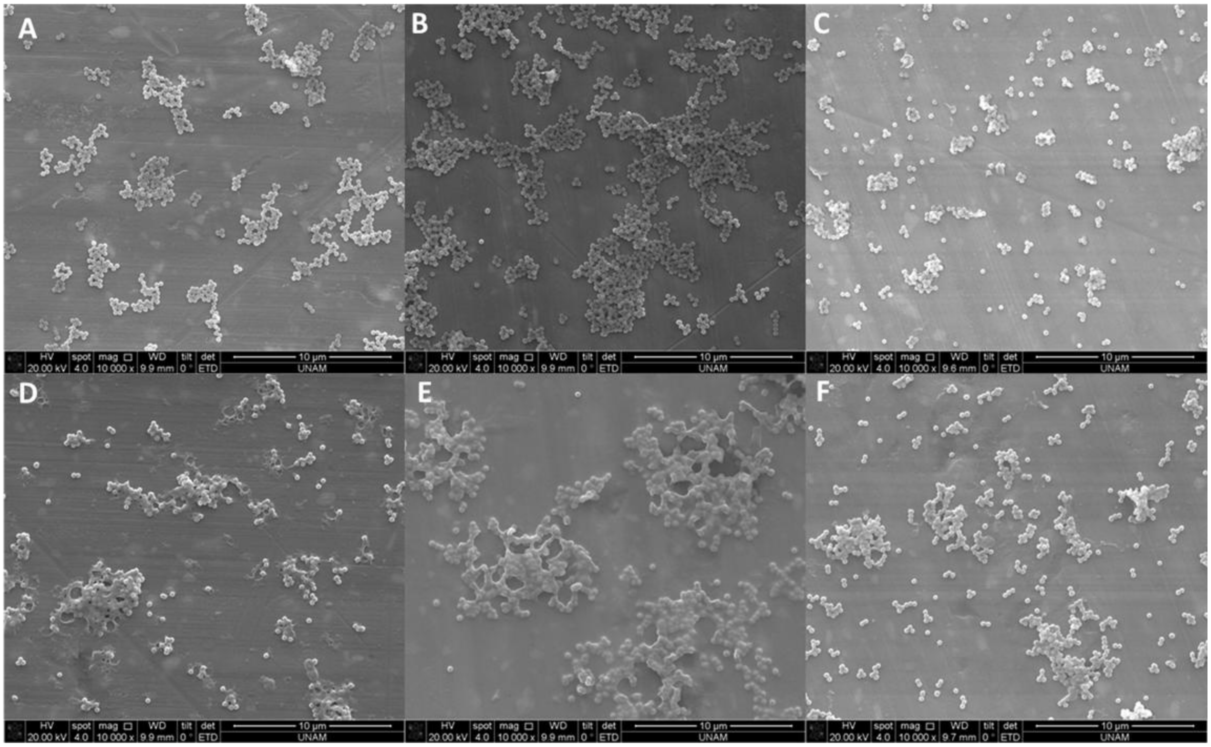

Figure S5. SEM image of digested bare Si300 NPs in A) SSF, (B) SGF, C) SIF and digested Si300 NPs pre-incubated with WM in D) SSF, E) SGF, F) SIF

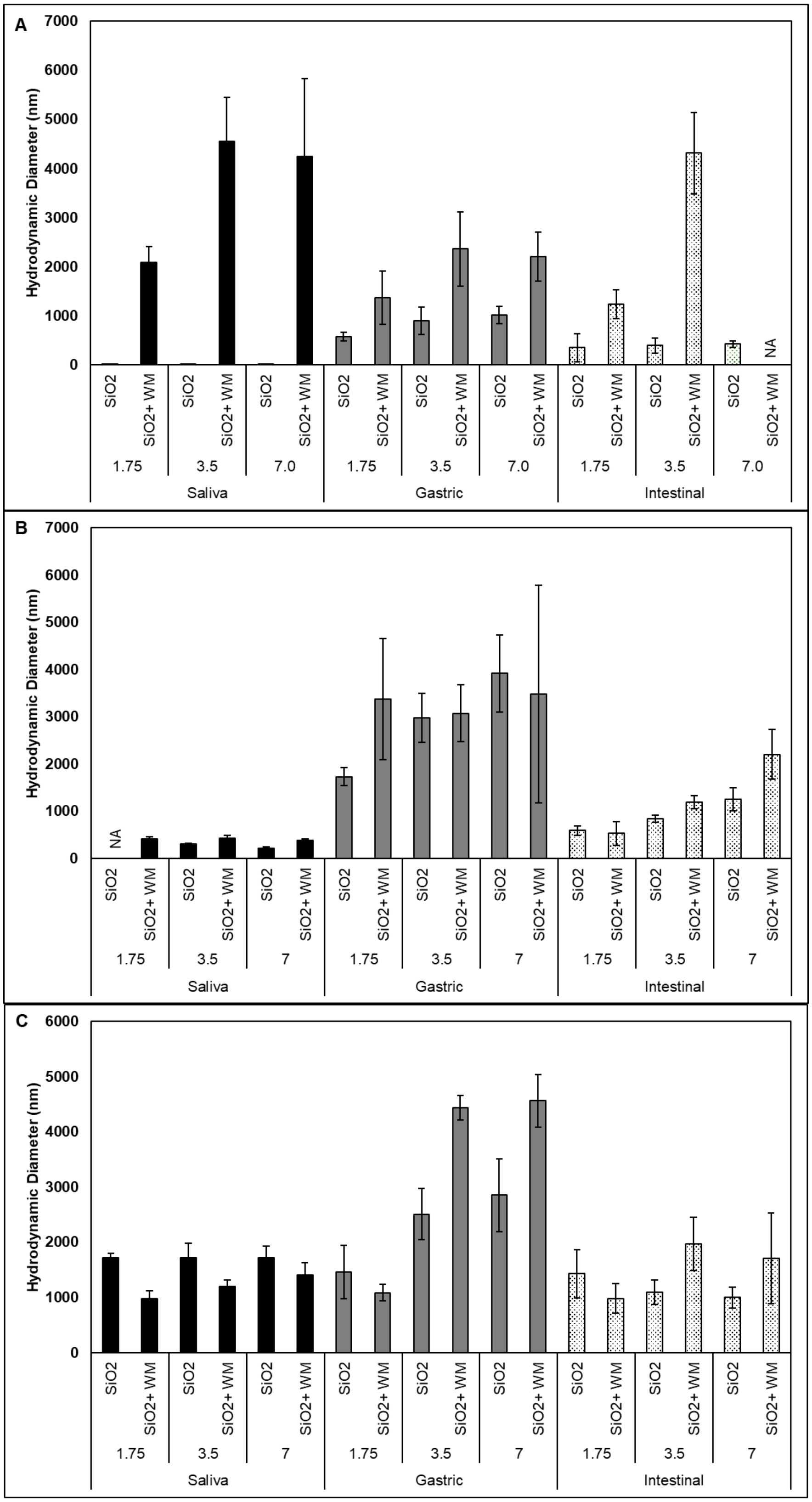

Figure S6. Average hydrodynamic diameters of A)Si25, B)Si100 and C)Si300 in saliva (black), gastric (grey) and intestine (dot) step of in vitro digestion simulation.

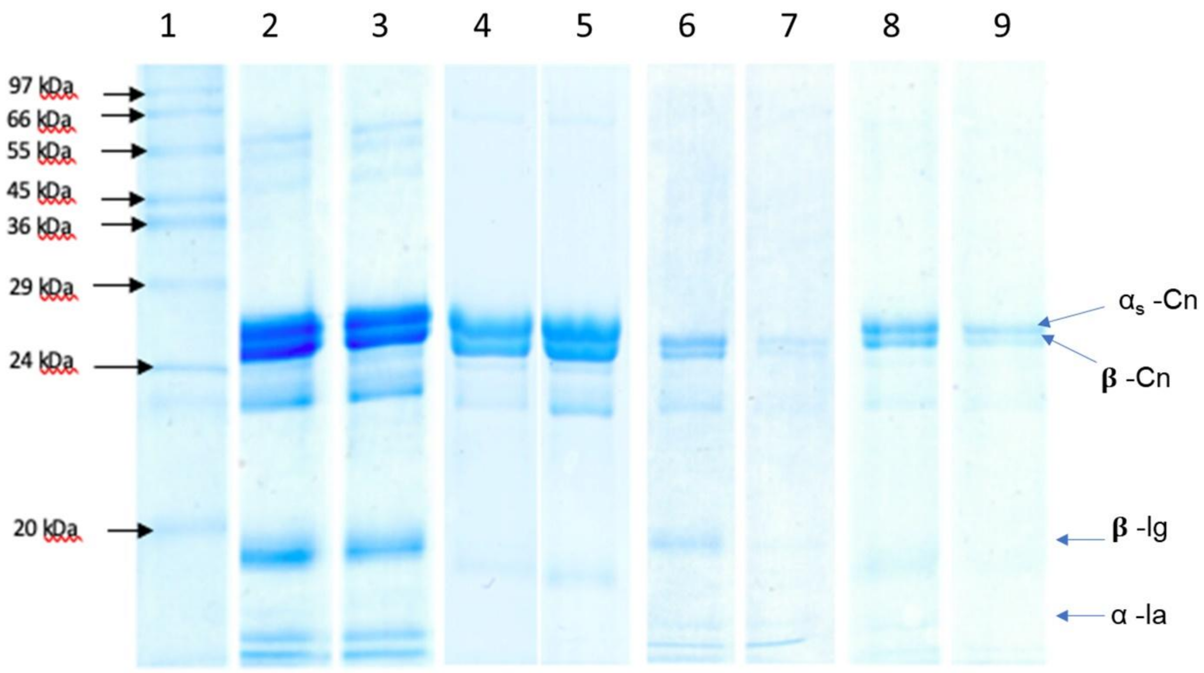

Figure S7. SDS-PAGE profile of SiO2 NPs interacted with milk samples. 1: Marker; 2: WM; 3: SM; 4: Si25+WM; 5: Si25+SM; 6: Si100+WM; 7:Si100+SM; 8: Si300+WM; 9: Si300+SM

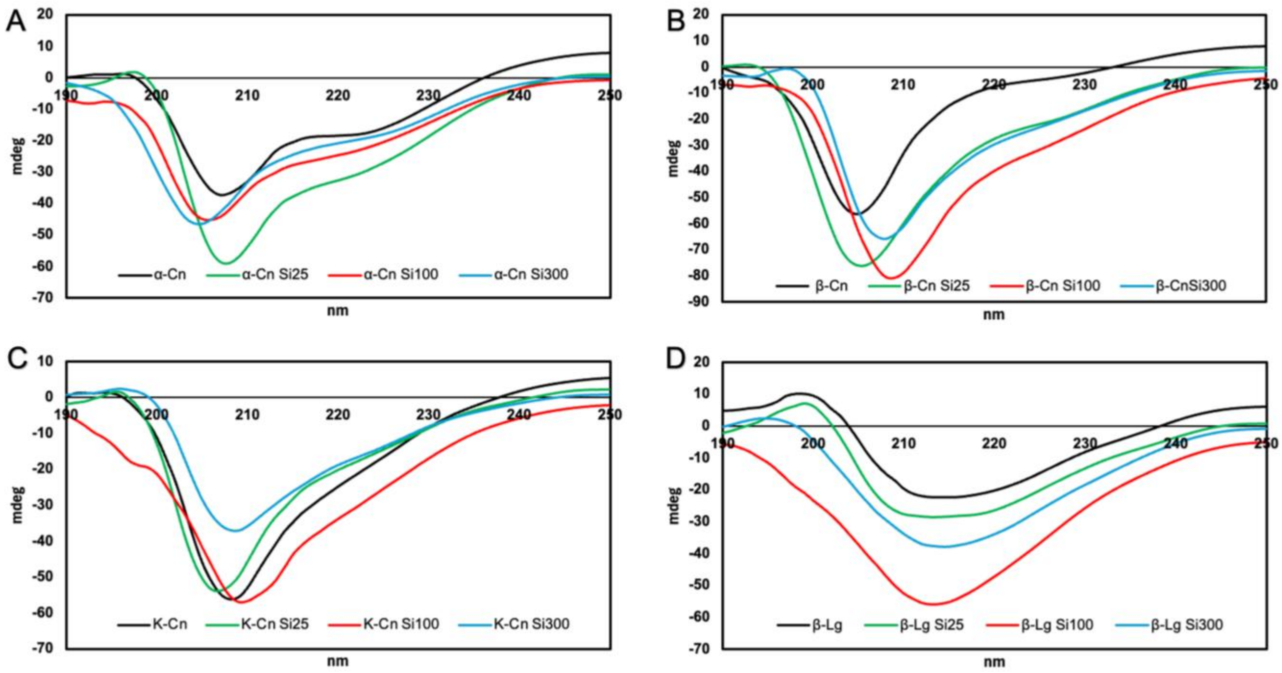

Figure S8. CD spectra of A) α-casein (α-Cn), B) β-casein (β-Cn), C) κ-casein (K-Cn), and D) β-lactoglobulin (β-Lg) in the absence and presence of SiO_2 NPs (Si25, Si100 and Si300).

Table S2. The average estimation of secondary structure fractions of α-casein, β-casein, κ-casein and β-lactoglobulin and after interaction with SiO_2 NPs (Si25, Si100 and Si300), with the usage of BeStSel online software.

	**α-helix**	**β-Sheet**	**Turn**	**Other**
**α-CN**	15.3	23.3	15.3	46.1
**α-CN + Si25**	17.4	16.0	18.7	47.9
**α-CN + Si100**	7.4	23.7	16.3	52.6
**α-CN + Si300**	6.4	25.9	15.9	51.8
**β-CN**	10.2	17.3	16.6	55.9
**β-CN + Si25**	13.7	19.2	15.7	51.4
**β-CN + Si100**	22.0	16.6	15.9	45.5
**β-CN + Si300**	19.9	13.5	19.1	47.5
**β-Lg**	7.8	34.6	15.1	42.5
**β-Lg + Si25**	6.8	32.9	13.3	47.0
**β-Lg + Si100**	4.5	32.1	11.7	51.7
**β-Lg + Si300**	4.3	34.9	11.6	49.2
**κ-CN**	19.2	19.0	14.1	47.7
**κ-CN + Si25**	14.9	19.3	15.3	50.5
**κ-CN + Si100**	13.2	25.0	13.3	48.5
**κ-CN + Si300**	11.2	27.4	14.1	47.3

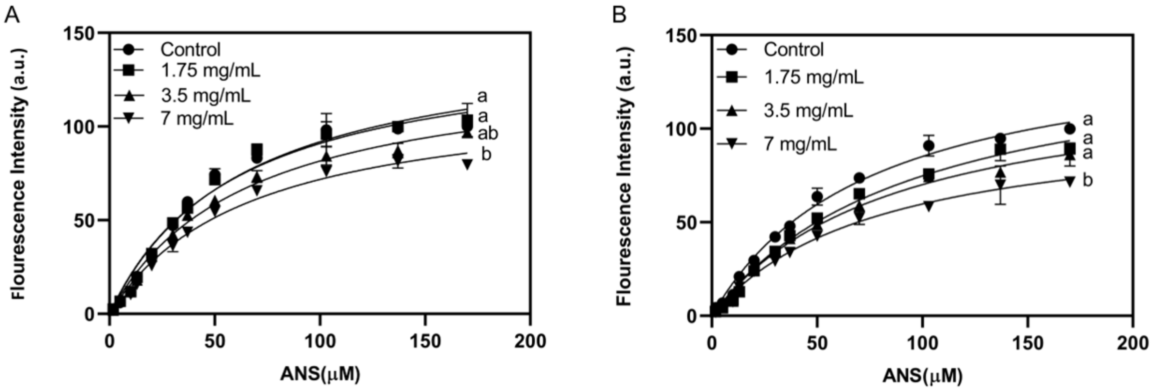

Figure S9. ANS titration curves of WM(A) and SM(B) in the absence and presence of Si25. Different letters indicate that significant differences (p<0.05) in the fluorescence intensity at the end point. The error bars represent mean ± S.D

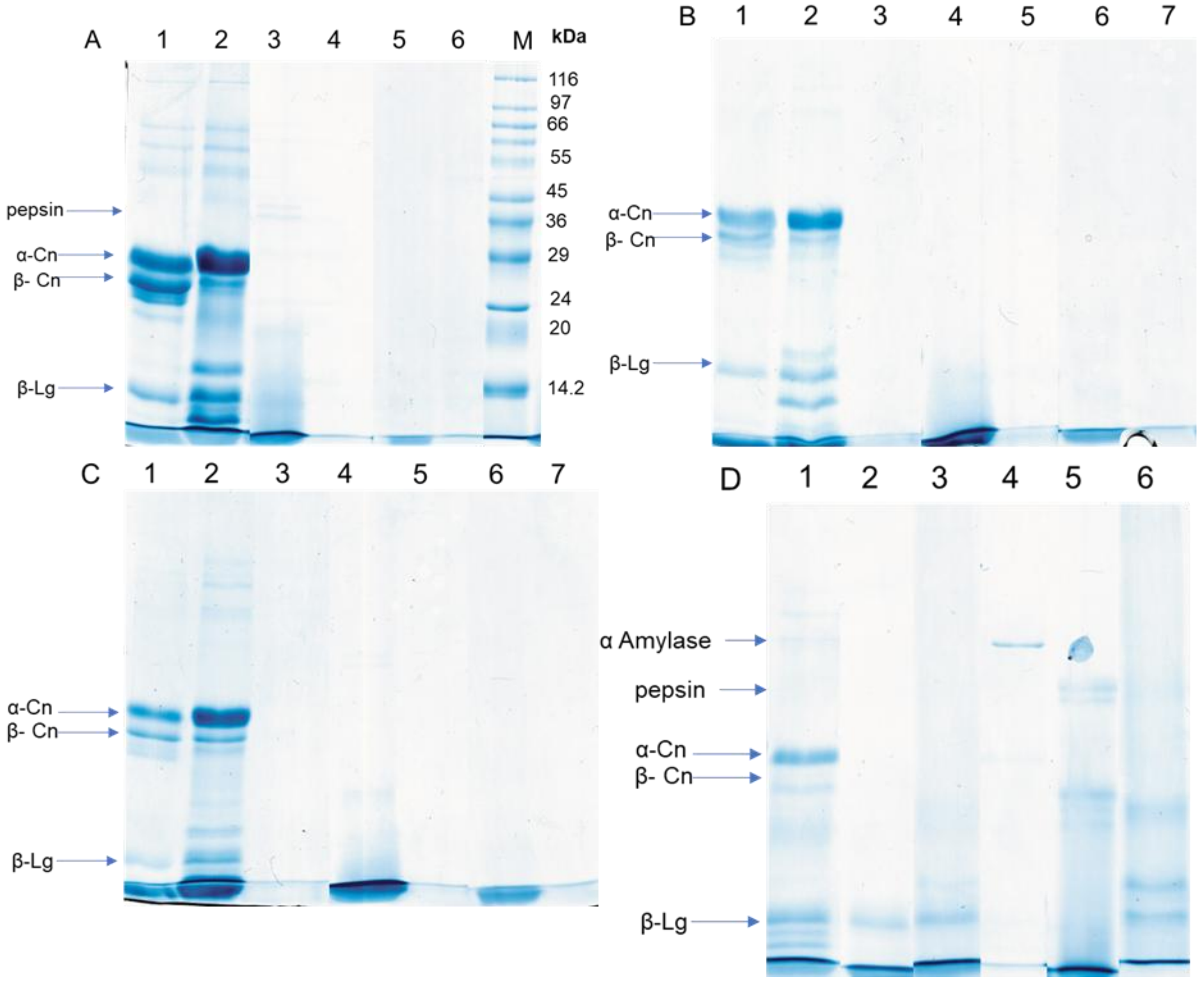

Figure S10. SDS-PAGE profiles of SiO2 NPs through the digestive system. A: Si25 (1:Si25+WM; 2: Si25+WM+SSF; 3: Si25+WM+SGF; 4: Si25+SGF; 5: Si25+WM+SIF; 6: Si25+SIF M: Marker); B: Si100 (1:

Si100+WM; 2: Si100+WM+SSF; 3: Si100+SSF; 4: Si100+WM+SGF; 5: Si100+SGF; 6: Si100+WM+SIF; 7: Si100+SIF); C: Si300 (1: Si300+WM; 2: Si300+WM+SSF; 3: Si300+SSF; 4: Si300+WM+SGF; 5: Si300+SGF; 6: Si300+WM+SIF; 7: Si300+SIF.); D: non-particle (*in-situ*) (1: WM+SSF; 2: WM+SGF; 3: WM+SIF; 4: SSF; 5: SGF; 6 SIF)

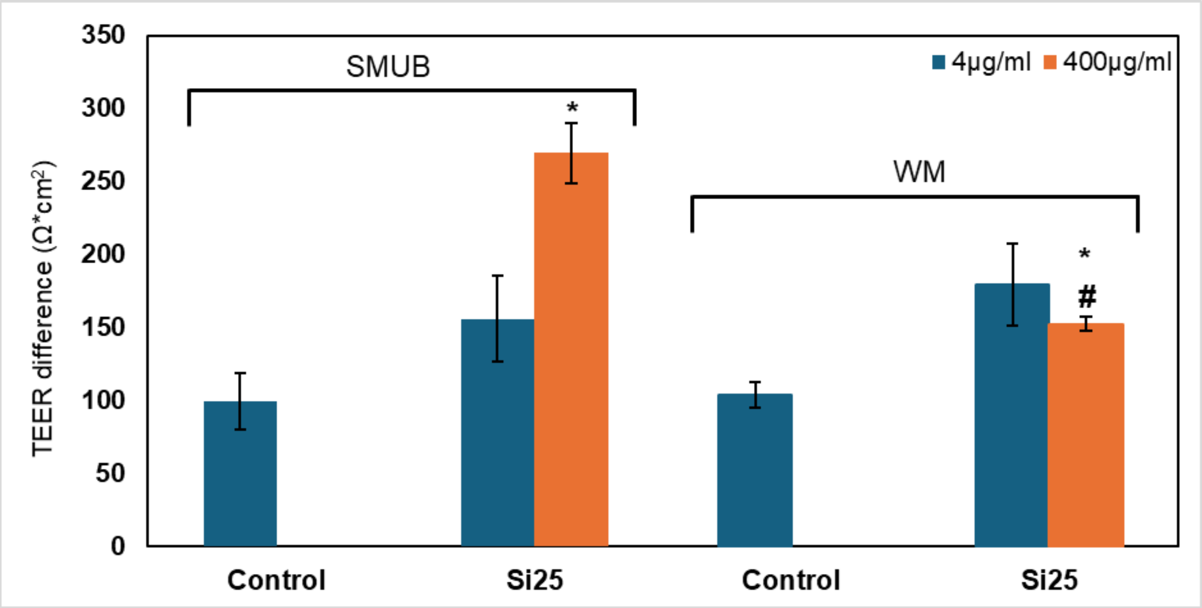

Figure S11. Average TEER change during the monolayer formation process (0-4 hour) of Caco-2 cells. * indicates that significant differences compared to control ($p<0.05$), # indicates that significant differences between WM incubated NPs and non-incubated (in SMUB) at same concentration ($p<0.05$).

REFERENCES

(1) Bao, N.; Miao, X.; Hu, X.; Zhang, Q.; Jie, X.; Zheng, X. Novel Synthesis of Plasmonic Ag/AgCl@TiO2 Continues Fibers with Enhanced Broadband Photocatalytic Performance. In *Catalysts*, 2017; Vol. 7.
